# Anatomical Bill

**Published:** 1853-05

**Authors:** 


*Anatomical Bill.* — We regret to learn that this bill, after having passed both branches of the legislature, was reconsidered, and finally killed in the senate. A senator who voted for it was induced to move a reconsideration, and to offer an amendment, which would cause it to be returned to the assembly, rendering its passage, owing to the late period in the session, impossible. Under these circumstances, the senate refused to adopt the amendment, and the senator referred to voted against the bill. The name of the senator has escaped us at the time we are writing, or we would give him the benefit of being known to our readers as the man who is responsible for the loss of the bill. The name shall appear in our columns hereafter.

